# Abnormal Default Mode Network Homogeneity in Major Depressive Disorder With Gastrointestinal Symptoms at Rest

**DOI:** 10.3389/fnagi.2022.804621

**Published:** 2022-03-30

**Authors:** Meiqi Yan, Jindong Chen, Feng Liu, Huabing Li, Jingping Zhao, Wenbin Guo

**Affiliations:** ^1^Department of Psychiatry, National Clinical Research Center for Mental Disorders, The Second Xiangya Hospital of Central South University, Changsha, China; ^2^Department of Radiology, Tianjin Medical University General Hospital, Tianjin, China; ^3^Department of Radiology, The Second Xiangya Hospital of Central South University, Changsha, China; ^4^Department of Psychiatry, The Third People’s Hospital of Foshan, Foshan, China

**Keywords:** major depressive disorder, default mode network, network homogeneity, gastrointestinal symptoms, magnetic resonance imaging

## Abstract

**Background:**

Gastrointestinal (GI) symptoms are prominent in many patients with major depressive disorder (MDD). However, it remains unclear whether MDD patients with GI symptoms have brain imaging alterations in the default mode network (DMN) regions.

**Methods:**

A total of 35 MDD patients with GI symptoms, 17 MDD patients without GI symptoms, and 28 healthy controls (HCs) were recruited. All participants underwent resting-state functional magnetic resonance imaging scans. Network homogeneity (NH) and support vector machine (SVM) methods were used to analyze the imaging data.

**Results:**

Gastrointestinal group showed higher 17-item Hamilton Rating Scale for Depression total scores and factor scores than the non-GI group. Compared with the non-GI group and HCs, the GI group showed decreased NH in the right middle temporal gyrus (MTG) and increased NH in the right precuneus (PCu). The SVM results showed that a combination of NH values of the right PCu and the right MTG exhibited the highest accuracy of 88.46% (46/52) to discriminate MDD patients with GI symptoms from those without GI symptoms.

**Conclusion:**

Major depressive disorder patients with GI symptoms have more severe depressive symptoms than those without GI symptoms. Distinctive NH patterns in the DMN exist in MDD patients with GI symptoms, which can be applied as a potential brain imaging marker to discriminate MDD patients with GI symptoms from those without GI symptoms.

## Introduction

Major depressive disorder (MDD) is a globally common, severe, and recurrent mental disorder (Smith, 2014). The World Health Organization (WHO) predicted that MDD will be the leading cause of the global burden of disease by 2030 (Malhi and Mann, 2018). However, the resources of professional psychiatric medical staff are scarce. Almost half of the whole world’s population lives in countries with only two professional psychiatrists per 100,000 people (Smith, 2014). A previous study has indicated that more healthcare resources were required during the episodes of MDD, resulting in a higher economic burden (Painchault et al., 2014). Comorbidity was reported as an important cause of the increasing economic burden of MDD (Greenberg et al., 2015). Many patients with MDD often have some non-specific somatic symptoms as their chief complaints [such as medically unexplained pain, insomnia, blurred vision, chest tightness, tachycardia, and gastrointestinal (GI) symptoms], with the prominent GI symptoms (such as loss of appetite, gastralgia, gastric distention, heartburn, acid reflux, nausea, vomiting, constipation, and diarrhea). In a large study of the general population, 54% of people with depressive symptoms suffered from frequent abdominal pain, constipation, diarrhea, dyspepsia, or irritable bowel syndrome (IBS), whereas only 29% of people with non-depressive symptoms (Hillilä et al., 2008). It has been indicated that somatic symptoms in MDD patients are associated with more severe clinical symptoms, lower remission rates (Novick et al., 2013), and worse prognosis (Bekhuis et al., 2016). Somatic symptoms also led to more utilization of health resources in patients with MDD (García-Campayo et al., 2008). In addition, patients with depressive-anxiety disorder comorbidities were reported to exhibit more several chronic physical conditions than patients without depression and anxiety comorbidities, and having depression and anxiety at the same time further increased the risk of a number of physical conditions co-occurring (Scott et al., 2007). Unfortunately, due to the scarcity of mental health resources, coupled with the social stigma of mental illness/disorders, MDD patients with somatic symptoms as chief complaints tend to seek treatment at general hospitals for the first time, even over and over again, leading to a lack of correct diagnosis and effective treatment (Kirmayer et al., 1993; García-Campayo et al., 2008), which is often called as “masked depression” (Verster and Gagiano, 1995). Thus, correct identifications and diagnoses of MDD patients with GI symptoms as chief complaints and effective treatments for them are urgent. It is not a one-way story that only patients with MDD can have GI symptoms. In clinical practice, patients with the digestive disease can also have psychological problems, such as anxiety, self-reported depressive mood, and even depression. A previous study has reported that most patients with GI motility disorders, IBS, and functional dyspepsia had a number of mental and psychological problems. When patients with IBS were exposed to stressors, their GI symptoms seem to be greatly increased (Whitehead, 1996). In another study, 44.4% of patients with inflammatory bowel disease (IBD) suffered from anxiety or depression or both, resulting in more therapies and healthcare resources (Navabi et al., 2018). IBD patients with baseline depression were reported to show more aggressive symptoms and poorer long-term progress than IBD patients without baseline depression (Kochar et al., 2018). Therefore, it is very important to find out the etiology of GI symptoms in patients with MDD.

But to date, the definite cause of MDD remains unknown. In recent years, the gut-brain axis has become a hot research topic, which is believed as one possible critical mechanism of affective disorders (Mayer et al., 2015). It has previously been reported that the GI microbiota can activate neural pathways and the central nervous system signaling systems, thereby affecting MDD-related symptoms (Foster and McVey Neufeld, 2013). However, it still remains unclear whether MDD patients with GI symptoms have brain imaging alterations. Structural (Bell-McGinty et al., 2002; Depping et al., 2016; Hyett et al., 2018) and functional (Bluhm et al., 2009; Guo et al., 2013b,Chen et al., 2015; Guo et al., 2018a) brain changes have already been reported in many previous studies of MDD. Meanwhile, some previous studies have reported structural and functional brain image changes in digestive system diseases (Kwan et al., 2005; Song et al., 2006; Blankstein et al., 2010; Skrobisz et al., 2020). Thus, some researchers tried to study whether MDD patients with GI symptoms exhibited abnormal brain imaging data and observed altered gray matter volume (GMV) and regional homogeneity (ReHo) (Yan et al., 2019; Liu et al., 2020). However, there are still few functional imaging studies conducted on MDD patients with GI symptoms, and the researchers did not further examine whether these abnormal GMV or ReHo could be used as good neuroimaging markers to discriminate the GI group from the non-GI group in both abovementioned studies.

The default mode network (DMN) is mainly composed of three subdivisions, namely, the ventral medial prefrontal cortex, the dorsal medial prefrontal cortex, the posterior cingulate cortex, and adjacent precuneus (PCu) plus the lateral parietal cortex (Raichle, 2015). Prior to this study, there have been many studies on the DMN of mental disorders, such as schizophrenia (Guo et al., 2014, 2015b; Hu et al., 2017; Hare et al., 2019; Fan et al., 2020; Martin-Subero et al., 2021), MDD (Guo et al., 2015a; Fang et al., 2016; Posner et al., 2016; Yan et al., 2019; Zhou et al., 2020), post-traumatic stress disorder (King et al., 2016; Miller et al., 2017; Viard et al., 2019), and attention deficit hyperactivity disorder (Sidlauskaite et al., 2016; Bozhilova et al., 2018). The increased amplitude of low-frequency fluctuation and functional connectivity (FC) in the DMN have been reported in patients with ulcerative colitis (Fan et al., 2019). Visceral sensory abnormalities are very common in IBS (Song et al., 2006). The DMN plays a critical role in gastric sensations (Skrobisz et al., 2020). A previous study has suggested that somatic symptom disorders may be associated with altered processing of sensory discrimination of pain and other somatic symptoms (Kim et al., 2019). It was indicated that brain areas involved in the process of pain sensory shifted to those involved in the subjective states of emotion and motivation in the majority of chronic pain diseases (Apkarian et al., 2011). Moreover, chronic visceral pain might lead to functional reorganization in the DMN (Farmer et al., 2012; Qi et al., 2016; Kano et al., 2018). Therefore, the abovementioned studies indicated that the DMN may change with the onset and development of GI symptoms. Thus, we were curious about whether there were special brain imaging changes of the DMN in these MDD patients with GI symptoms.

In this study, we applied a network homogeneity (NH) approach to examine whether the NH of the DMN in MDD patients with GI symptoms was abnormal and whether abnormal NH of the DMN could be used as brain imaging markers to separate MDD patients with GI symptoms from MDD patients without GI symptoms. We hypothesized that MDD patients with GI symptoms exhibited altered NH in certain areas of the DMN, which might be used to discriminate the MDD patients with GI symptoms from those without GI symptoms.

## Materials and Methods

### Participants

A total of 35 patients with at least one GI symptom were recruited as the GI group, and 17 patients without GI symptoms were recruited and assigned to the non-GI group. Main GI symptoms included medically unexplained gastralgia, gastric distention, heartburn, acid reflux, nausea, vomiting, constipation, and diarrhea. All patients were aged from 18 to 55 years and were from the outpatients of the Second Xiangya Hospital of Central South University, China. Their final diagnoses were independently confirmed by two psychiatrists, using the DSM-5 criteria for MDD. The inclusion criteria for all patients were as follows: (1) first major depressive episode; (2) 17-item Hamilton Rating Scale for Depression (HRSD-17) (Hamilton, 1967) total scores ≥18; (3) with no history of antipsychotics and physical therapy (such as electroconvulsive therapy, ECT); and (4) with no confirmed digestive diseases.

A total of 28 healthy controls (HCs) were recruited from the community; the patient groups were matched in age, gender, and education. They would not be recruited if they had a suspicious or explicit family history of mental illness/disorders, especially their first-degree relatives. Besides, HCs with any history of digestive diseases, neurological diseases, substance abuse, or psychotic symptoms would not be enrolled.

All participants were Han Chinese and right-handed. The exclusion criteria for all participants were as follows: (a) other mental disorders meeting DSM-5 diagnostic criteria; (b) any history of neurological diseases, severe physical illnesses, and substance abuse; (c) being pregnant; (d) structural abnormalities of the brain after the initial magnetic resonance imaging (MRI) scan; and (e) any contraindications to MRI scans.

The severity of MDD was evaluated by using the HRSD-17. It can be divided into five types of factors: (1) anxiety/somatization (six items, namely, psychic anxiety, somatic anxiety, GI symptoms, hypochondriasis, insight, and general symptoms); (2) weight loss (one item); (3) cognitive disturbances (three items, namely, self-guilt, suicide, and agitation); (4) retardation symptoms (four items, namely, depression, work and interests, retardation, and sexual symptoms); and (5) sleep disturbances (three items, namely, difficulty in falling asleep, superficial sleep, and early awakening).

The study was approved by the Medical Research Ethics Committee of the Second Xiangya Hospital of Central South University, China. It was conducted in accordance with the Helsinki Declaration. Each participant has submitted a written informed consent before enrollment.

### Image Acquisition

By using a 3.0 T Siemens scanner (Germany) at the Second Xiangya Hospital of Central South University, resting-state fMRI data were obtained. All subjects were instructed to close their eyes, stay still, and awake throughout the scan. The resting-state functional images were acquired using the echo-planar imaging sequence. Specific parameters were as follows: 2,000/30 ms of repetition time/echo time, 30 slices, 64 × 64 matrix, 90° flip angle, 24 cm field of view, 4 mm slice thickness, 0.4 mm gap, and 250 volumes lasting for 500 s.

### Data Preprocessing

A Data Processing Assistant for Resting-State fMRI was used for data preprocessing in MATLAB (MathWorks) (Chao-Gan and Yu-Feng, 2010). Considering that data errors may increase due to initial signal instability and subjects’ early adaptation time, we deleted the first 10 original images to minimize these potential impacts. Specific processes were as follows: (a) correction for slice timing and head motion: all data were with a maximum displacement of *x*-, *y*-, or *z*-axis less than 2 mm and maximum angular rotation less than 2°; (b) normalization: the corrected imaging data then got spatially normalized to the MNI space with 3 mm × 3mm × 3 mm; and (c) band-pass filtering and detrending: after normalization, the imaging data were temporally band-pass filtered at 0.01–0.08 Hz and got linearly detrended. Several spurious covariates (such as the signal from the ventricular seed-based region of interest, white matter-centered region, and 24-head motion parameters acquired by rigid body correction) were removed from the imaging data. The global signal was not regressed out during data preprocessing according to a previous study (Hahamy et al., 2014).

### Default Mode Network Identification

After being preprocessed, all groups were subjected to construct the DMN mask by using Group Independent Component Analysis (ICA) of fMRI toolbox (GIFT).^1^ Three main steps were included in the analysis as follows: data reduction, independent component separation, and back reconstruction. The DMN components were selected based on the templates provided by GIFT. The specific calculation process of generating the final DMN mask was similar to our previous study (Guo et al., 2018b). The generated DMN mask was applied in the following NH analysis.

### Network Homogeneity Analysis

The NH analysis was conducted using MATLAB (MathWorks). Correlation coefficients between each voxel and all other voxels within the DMN mask were calculated for each subject. The average correlation coefficient was defined as the homogeneity of a given voxel. The NH of each voxel in the DMN mask was generated. The NH maps were smoothed with a Gaussian kernel of 4 mm full width at half maximum and were used for group comparisons.

### Statistical Analyses

Group differences (such as age, years of education, HRSD-17 total, and the five-factor scores across the three groups) were compared by performing an analysis of variance (ANOVA) using SPSS19.0 (LSD between two group comparisons). A Chi-square test was applied to describe gender distribution. And we applied a two-sample *t*-test to analyze whether there were differences in the illness duration between the GI and non-GI groups. The *p*-value of <0.05 was considered statistically significant.

The NH analysis was performed using an analysis of covariance (ANCOVA) across the three groups, followed by *post hoc t*-tests. The significance threshold for multiple comparisons was set at *p* < 0.05 by using Gaussian random field (GRF) theory (voxel significance at *p* < 0.001 and cluster significance at *p* < 0.05). Frame-wise displacement (FD) values were calculated for every subject, and the average FD was used as one of the covariates according to the previous study (Power et al., 2012). Age, sex, and years of education were other covariates.

### Correlation Analyses

The NH values were extracted from the brain clusters with abnormal NH values. Pearson’s correlation analysis was used to determine the correlations between NH abnormality and HRSD-17 total scores as well as the five-factor scores with the Benjamini–Hochberg correction threshold of *p* < 0.05.

### Classification Analyses

The support vector machine (SVM) approach is a popular supervised machine-learning model, which has gotten many applications for classifications in the researches of psychiatry in recent years (Steardo et al., 2020). It can map training examples to points in space and construct a hyperplane with the maximum distance from the nearest training data point of any one of the two predefined categories, known as the maximum-margin hyperplane (Shan et al., 2020). These nearest points are support vectors (Du et al., 2015). For more details about SVM, a previous study (Shan et al., 2020) is available. In this study, SVM was applied to use NH values extracted from the brain regions with abnormal NH values to discriminate MDD patients with GI symptoms from those without GI symptoms by employing the library for SVM (LIBSVM) software package^2^ in MATLAB. In this study, the method of “leave-one-out” was applied.

## Results

### Demographic Characteristics and Clinical Information

The three groups did not show any significant group differences in age, years of education, and gender distribution. No significant illness-duration difference was observed between the two patient groups. Both patient groups showed higher HRSD-17 total and factor scores (except for scores of weight loss) than HCs. The weight loss scores of the GI group were higher than those of both the non-GI group and HCs, but there was no significant difference in weight loss scores between the non-GI group and HCs. Besides, the GI group showed higher HRSD-17 total scores, factor scores of anxiety/somatization, and sleep disturbances than those of the non-GI group (Table 1).

**TABLE 1:** Demographic and clinical characteristics of the participants.

	S1 (*n* = 35)	S0 (*n* = 17)	HCs (*n* = 28)	*F*, *t* or χ ^2^ value	*Post hoc t*-tests or *p*-values
Age (years)	30.86 ± 6.84	30.29 ± 8.05	30.14 ± 5.00	0.102	0.903*^a^*
Gender (male/female)	13/22	6/11	14/14	1.377	0.502*^b^*
Handedness (right/left)	35/0	17/0	28/0		
Education (years)	14.51 ± 3.28	12.94 ± 3.46	14.61 ± 2.69	1.797	0.173*^a^*
Illness duration (months)	6.23 ± 4.63	6.94 ± 3.98		0.544	0.589*^c^*
HRSD-17 scores	22.69 ± 3.41	20.18 ± 2.67	0.89 ± 0.88	585.979	S1 > S0 > Nor
Anxiety/somatization	7.31 ± 1.92	6.41 ± 1.66	0.39 ± 0.57	174.531	S1 > S0 > Nor
Weight loss	0.80 ± 0.83	0.06 ± 0.24	0	18.741	S1 > S0, Nor
Cognitive disturbances	3.71 ± 1.78	3.41 ± 1.50	0	64.213	S1, S0 > Nor
Retardation symptoms	6.40 ± 1.42	6.76 ± 1.56	0.18 ± 0.39	253.030	S1, S0 > Nor
Sleep disturbances	4.46 ± 1.42	3.53 ± 1.28	0.32 ± 0.55	103.570	S1 > S0 > Nor

*HRSD-17, 17-item Hamilton Rating Scale for Depression.*

*^a^The p-value was obtained by analyses of variance.*

*^b^The p-value was obtained by a Chi-square test.*

*^c^The p-value was obtained by two-sample t-tests.*

*S1, gastrointestinal (GI) symptoms group.*

*S0, non-gastrointestinal (non-GI) symptoms group. HCs, healthy controls.*

### Network Homogeneity Differences Across Groups

**FIGURE 1:**
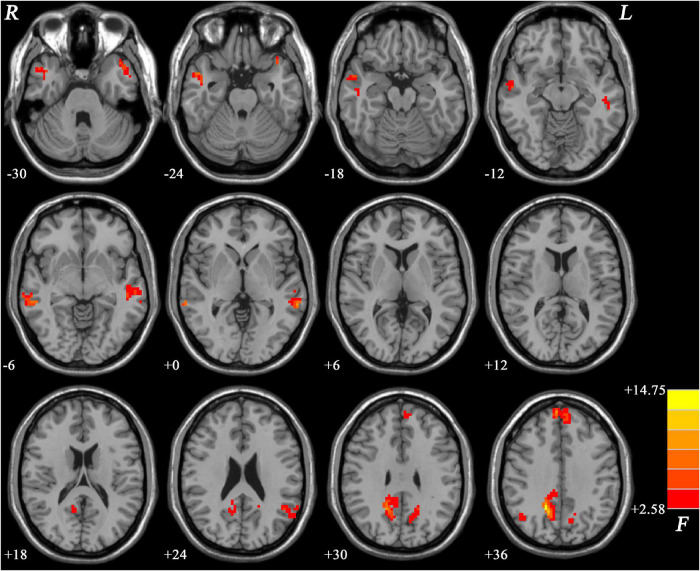
Brain regions within the DMN showing group differences in NH values across the three groups. Color bar indicates *F* values from ANCOVA (age, sex, years of education, and frame-wise displacement as covariates). NH, network homogeneity; DMN, default mode network; ANCOVA, analysis of covariance.

The NH values showed significant differences mainly in the frontal and temporal regions across the three groups (Figure 1).

**FIGURE 2:**
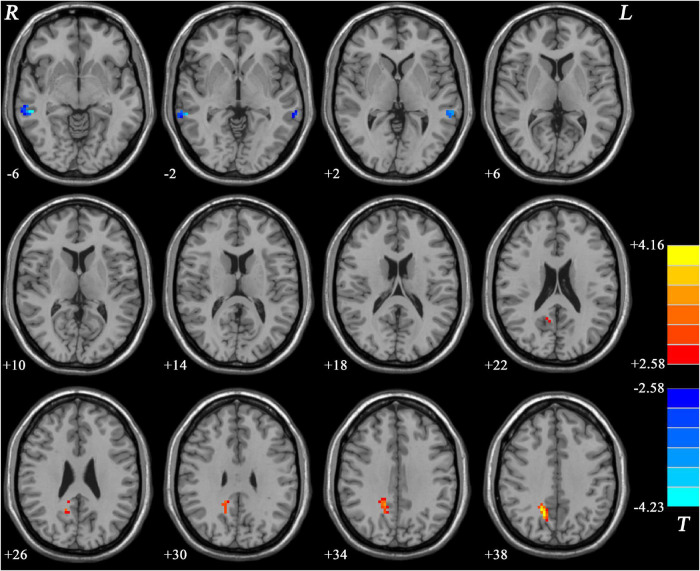
Statistical map depicts higher and lower NH of MDD patients with GI symptoms compared with MDD patients without GI symptoms. The threshold was set at *p* < 0.05. Blue denotes lower NH, and red denotes higher NH. Color bar indicates *T* values from the two-sample *t*-test. NH, network homogeneity; GI, gastrointestinal symptoms.

**FIGURE 3:**
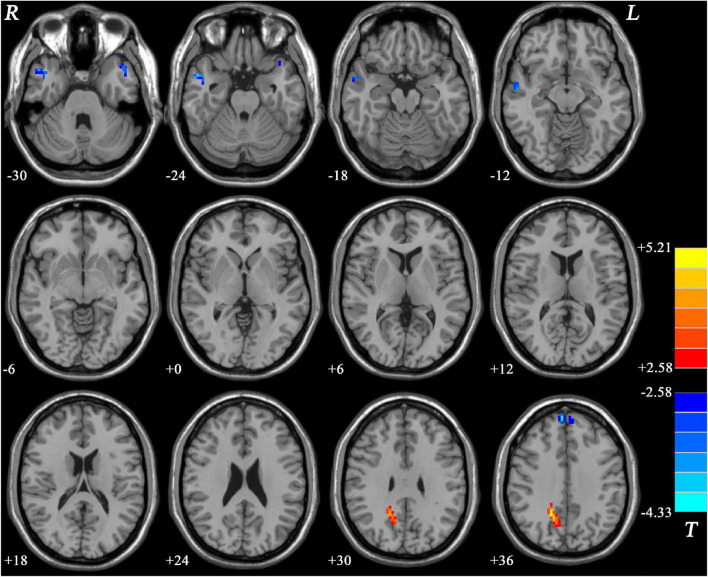
Statistical map depicts higher and lower NH of MDD patients with GI symptoms compared with healthy controls. The threshold was set at *p* < 0.05. Blue denotes lower NH, and red denotes higher NH. Color bar indicates *T* values from two-sample *t*-test. NH, network homogeneity; GI, gastrointestinal symptoms.

Compared with MDD patients without GI symptoms, MDD patients with GI symptoms showed decreased NH in the bilateral MTG and increased NH in the right PCu (Figure 2 and Table 2). Compared with HCs, MDD patients with GI symptoms exhibited decreased NH in the bilateral superior medial frontal cortex, left middle temporal pole, right MTG, and increased NH in the right PCu (Figure 3 and Table 2). No abnormal NH in any brain region was found in the non-GI group relative to HCs.

**TABLE 2:** Significant NH differences of DMN across groups.

Cluster location	Peak (MNI)			Number of voxels	*T* value
	*x*	*y*	*z*		
**S1 vs. S0**					
Right middle temporal gyrus	54	−36	−6	26	−4.2276
Left middle temporal gyrus	−63	−39	3	20	−3.5664
Right precuneus	15	−57	39	88	4.1589
**S1 vs. HC**					
Bilateral superior medial frontal cortex	6	57	36	35	−4.1248
Left middle temporal pole	−45	15	−30	24	−3.4925
Right middle temporal gyrus	45	3	−27	52	−4.3309
Right precuneus	18	−54	36	76	5.2115
**S0 vs. HCs**					
None					

*NH, network homogeneity.*

*DMN, default mode network.*

*MNI, Montreal Neurological Institute.*

*S1, gastrointestinal (GI) symptoms group.*

*S0, non-gastrointestinal (non-GI) symptoms group.*

*HCs, healthy controls.*

### Support Vector Machine Analysis in Major Depressive Disorder With and Without Gastrointestinal Symptoms

**FIGURE 4:**
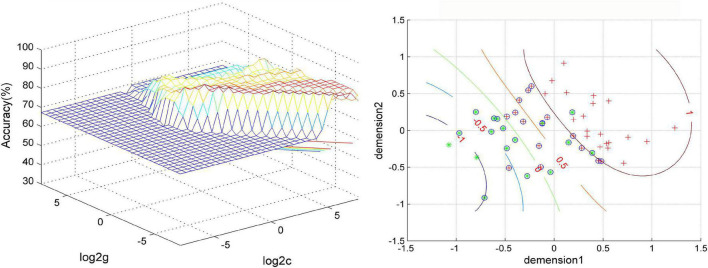
Visualization of classifications through support vector machine (SVM) using a combination of NH values in the right precuneus and right middle temporal gyrus. Left: SVM parameters result of 3D view. Right: dimension 1 and dimension 2 represent the NH values in the right precuneus and right middle temporal gyrus, respectively. Red crosses represent MDD patients with GI symptoms, and green crosses represent MDD patients without GI symptoms. The circles mean support vectors. MDD, major depressive disorder; GI, gastrointestinal symptoms.

**FIGURE 5:**
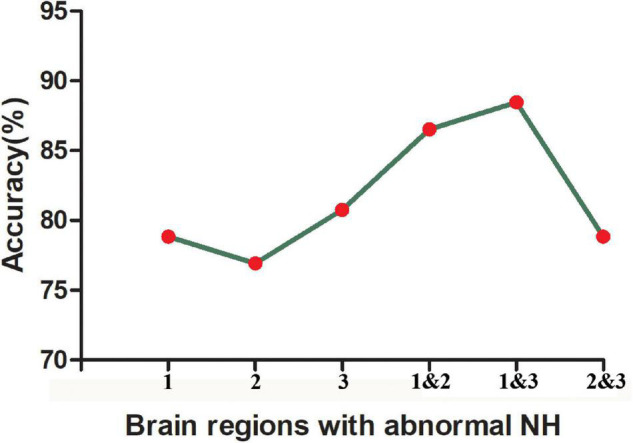
The accuracy of using abnormal NH values of different brain regions to classify two patient groups. 1, right precuneus; 2, left middle temporal gyrus; 3, right middle temporal gyrus; 1&2, right precuneus and left middle temporal gyrus; 1&3, right precuneus and right middle temporal gyrus; 2&3, left middle temporal gyrus and right precuneus and right middle temporal gyrus.

The SVM results showed that a combination of NH values of the right PCu and the right MTG exhibited the highest accuracy of 88.46% (46/52) to discriminate MDD patients with GI symptoms from those without GI symptoms, with a sensitivity and specificity of 97.14% (34/35) and 70.56% (12/17), respectively. The accuracy of using abnormal NH in different brain regions was 78.85% (41/52) of the right PCu, 76.92% (40/52) of the left MTG, 80.77% (42/52) of the right MTG, 86.54% (45/52) of a combination of the right PCu and the left MTG, and 78.85% (41/52) of a combination of the bilateral MTG (Figure 4 and Figure 5).

### Correlations Between Network Homogeneity and Clinical Characteristics

There was no significant correlation between abnormal NH and the HRSD-17 total scores as well as the five-factor scores for both GI and non-GI symptom groups.

## Discussion

Major depressive disorder patients with GI symptoms showed greater severity of depressive symptoms than MDD patients without GI symptoms. Distinctive NH patterns existed in MDD patients with GI symptoms. Compared with the non-GI group and HCs, the GI group showed decreased NH in the right MTG and increased NH in the right PCu. SVM results showed that a combination of NH values of the right PCu and the right MTG might be a potential brain imaging marker to discriminate MDD patients with GI symptoms from those without GI symptoms.

Depression, anxiety, and other psychological factors were considered as one of the important causes or inducements of some digestive system diseases (Drossman et al., 1999; Fond et al., 2014; Lee et al., 2017). A previous study has reported more serious depression in MDD patients with GI symptoms (Liu et al., 2020). Consistent with it, we observed that the GI group showed higher HRSD-17 total scores, factor scores of anxiety/somatization, weight loss, and sleep disturbances than the non-GI group in this study, indicating that the GI symptoms may be related to more severe depression.

The temporal gyrus is involved in attention control with the frontal and parietal lobe together (Sani et al., 2021), in which the MTG participated in cued attention (Corbetta and Shulman, 2002). Our previous study has observed that melancholic MDD showed lower NH in the right MTG than HCs (Cui et al., 2017). Reduced gray matter (Peng et al., 2011; Ma et al., 2012) and abnormal functional network connectivity (Zhi et al., 2018) were also observed in the MTG in MDD compared with HCs. Furthermore, a previous study found that MDD patients with somatic symptoms showed lower ReHo in the right MTG compared with MDD patients without somatic symptoms (Geng et al., 2019). In line with these studies, we observed decreased NH in the right MTG in MDD patients with GI symptoms compared with both MDD patients without GI symptoms and HCs. These issues might explain the phenomenon that MDD patients with GI symptoms pay much more attention to their somatic symptoms. Thus, we suspected that the right MTG might play an important role in the pathophysiology of MDD with GI symptoms. Additionally, we observed increased NH in the right PCu in MDD patients with GI symptoms compared with both MDD patients without GI symptoms and HCs. Some previous studies have reported abnormal PCu in MDD, including higher activity (Sheline et al., 2010) and lower activity (Chen et al., 2012; Guo et al., 2013a; Shi et al., 2020). It was proposed that PCu was involved in consciousness, specifically in the processes of self-reflection and episodic memory retrieval (Cavanna and Trimble, 2006; Cavanna, 2007). PCu would selectively deactivate during sleep (Cavanna and Trimble, 2006; Cavanna, 2007). Thus, we suspected that abnormal NH in the right PCu might be correlated with the phenomenon that the GI group showed more severe sleep disturbance than the non-GI group. Unfortunately, we did not find any statistically significant correlation between abnormal NH in the right PCu and clinical features. It was inconsistent with the results of the abovementioned study since we recruited different types of patients with MDD. Interestingly, a previous study reported that the interregional FC of the DMN between the MTG and PCu was reduced in IBS patients compared with HCs (Qi et al., 2016). Although we did not obtain more interregional FC data here to further test it, it makes us sure that decreased NH in the right MTG and increased NH in the right PCu might be distinctive NH patterns in MDD with GI symptoms.

We performed the SVM analysis to test whether abnormal NH in the DMN region could be used as a brain imaging marker to screen MDD patients with GI symptoms from MDD patients without GI symptoms. The results showed that a combination of NH values of the right PCu and right MTG showed the highest accuracy of 88.46% (46/52) to discriminate MDD patients with GI symptoms from those without GI symptoms, with a sensitivity of 97.14% (34/35) and a specificity of 70.56% (12/17). The establishment of good diagnostic indicators requires sensitivity or specificity of at least 60%, preferably greater than 70% (Swets, 1988; Gong et al., 2011). Thus, the combination of NH values of the right PCu and right MTG may be a potential brain imaging marker to screen MDD patients with GI symptoms from MDD patients without GI symptoms.

There were some limitations. First, we did not evaluate the severity of GI symptoms and did not further classify them. Second, we did not know whether changes in NH occurred before or as a result of GI symptoms. A long-term follow-up observation in the non-GI group may help us to understand the cause and effect. If the NH changes are prior, we could use the neuroimaging marker to identify patients who may have GI symptoms in advance in the future, provide appropriate intervention to prevent more serious symptoms, and then save medical resources. On the contrary, if abnormal NH is the result of GI symptoms, it will provide a new research direction for related treatments.

## Conclusion

Major depressive disorder with GI symptoms shows more severe depressive symptoms than MDD without GI symptoms. Distinctive NH patterns in DMN exist in MDD with GI symptoms that can be applied as a potential brain imaging marker to discriminate MDD with GI symptoms from those without GI symptoms.

## Data Availability Statement

The raw data supporting the conclusions of this article will be made available by the authors, without undue reservation.

## Ethics Statement

The studies involving human participants were reviewed and approved by the Medical Research Ethics Committee of the Second Xiangya Hospital of Central South University, China. The patients/participants provided their written informed consent to participate in this study.

## Author Contributions

MY, HL, and WG contributed to the conception and design of the study. HL, JC, and JZ supervised the progress of the study. MY, FL, and WG performed the data analysis. MY wrote the manuscript. All authors contributed to manuscript revision, read, and approved it for publication.

## Conflict of Interest

The authors declare that the research was conducted in the absence of any commercial or financial relationships that could be construed as a potential conflict of interest.

## Publisher’s Note

All claims expressed in this article are solely those of the authors and do not necessarily represent those of their affiliated organizations, or those of the publisher, the editors and the reviewers. Any product that may be evaluated in this article, or claim that may be made by its manufacturer, is not guaranteed or endorsed by the publisher.

## Footnotes

1
http://mialab.mrn.org/software/#gica

2
http://www.csie.ntu.edu.tw/~cjlin/libsvm/
